# Effect of hematuria on the outcome of immunoglobulin A nephropathy with proteinuria

**DOI:** 10.15171/jnp.2016.12

**Published:** 2016-02-25

**Authors:** Chihiro Iwasaki, Takahito Moriyama, Kayu Tanaka, Takashi Takei, Kosaku Nitta

**Affiliations:** ^1^Department of Medicine, Kidney Center, Tokyo Women’s Medical University, Tokyo, Japan

**Keywords:** IgA nephropathy, Hematuria, Urinary red blood cells, Proteinuria, Renal function

## Abstract

*Background:* The relationship between hematuria and histological lesions, the effect of hematuria on response to steroid therapy, and the outcome in patients with immunoglobulin A nephropathy (IgAN) remain undetermined.

*Objectives:* The aim of this study was to clarify the effect of hematuria on histological findings, response to steroid treatment, and the outcome in IgA nephropathy.

*Patients and Methods:* Seventy-five patients with IgAN and proteinuria > 1 g/day and treated with prednisolone were divided into two groups: those with low (≤20/high-power field [HPF]) urinary red blood cell (U-RBC) counts (L-RBC group, n=55) and those with high (>20/HPF) U-RBC counts (H-RBC group, n=20). Their clinical and histological characteristics, the relationship between hematuria and histological lesions, renal outcomes, and risk factors for progression were compared.

*Results:* Except for U-RBC counts, the clinical and histological findings according to the Oxford classification of the two groups were similar. U-RBC counts were not correlated with active histological lesions. Median proteinuria in both groups decreased soon after starting steroid therapy. Median U-RBC also decreased after starting steroids, and it became similar between both groups at 2 years after treatment. The 20-year renal survival rate was also similar between the H-RBC and the L-RBC group (45.2% versus 58.0%, P=0.5577). Multivariate Cox regression analysis showed that the lower estimated glomerular filtration rate (eGFR) was an independent risk factor for progression.

*Conclusions:* A higher degree of hematuria at renal biopsy in patients with IgAN was not associated with active pathological lesions, such as cellular and fibro-cellular crescents, resistance to steroid treatment and poor outcome.

**Implication for health policy/practice/research/medical education:**

In a study on 75 patients with IgAN and proteinuria >1 g/day, we found, the degree of hematuria did not have any relationship with histological activity. Additionally, the effect of steroid treatment was similar and long-term prognosis after steroid treatment was also similar, regardless of the degree of hematuria.

## 1. Background

The degree of proteinuria was found to be the optimal surrogate marker of progression to end-stage renal disease (ESRD) during the treatment of patients with immunoglobulin A nephropathy (IgAN). In contrast, the level of hematuria was still controversial to evaluate the effect of treatment in patients with IgAN. Therefore, many studies showed serial changes in proteinuria and estimated glomerular filtration rate (eGFR) during follow-up, but few studies showed the degree of hematuria. In 2001, however, tonsillectomy combined with steroid pulse therapy was shown to decrease both hematuria and proteinuria, with none of these patients progressing to ESRD over a 10-year follow-up period (1). Since then, IgAN patients in Japan have been more frequently treated with tonsillectomy and steroid pulse therapy (2,3), with the effectiveness of these treatments determined by monitoring the degrees of both hematuria and proteinuria (4-6). Although several investigators emphasized that hematuria has been regarded as proof of inflammation of glomerular capillaries, it has been still unknown whether the degree of hematuria was absolutely reflected of the severity of capillaritis, such as endocapillary hypercellularity, lysis of capillaries, cellular and fibro-cellular crescents. Moreover, it has also still unknown whether the prognosis of IgAN patients with severe hematuria would be worse than that of patients with mild hematuria, with the degree of hematuria having a negative impact on response to steroid treatment.

We previously reported that, in IgAN patients with mild proteinuria (less than 0.5 g/day), the hematuria was naturally decreased without any intensive therapy and severe hematuria was not related with the progression to increasing proteinuria and ESRD (7). To investigate the whole effect of hematuria on IgAN, this study was designed to evaluate the correlation between hematuria and histological findings, the effect of hematuria on response to steroid treatment, and the long-term renal survival of IgAN patients with severe proteinuria (over 1.0 g/day).

## 2. Objectives

The aim of present study was to clarify whether the severe hematuria have relationship with histological background, response to steroid treatment and prognosis in proteinuric IgAN.

## 3. Patients and Methods

### 3.1. Patients

Of 743 patients diagnosed with IgAN by renal biopsy at Tokyo Women’s Medical University from 1980 to 2005, 75 had proteinuria greater than 1g/day, had been treated with prednisolone, and were followed up for at least 1 year after renal biopsy. These 75 patients were divided into two groups: those with low (≤20/high-power field [HPF]) urinary red blood cell (U-RBC) counts (L-RBC group, n=20) and those with high (>20/HPF) U-RBC counts (H-RBC group, n=55). We decided the boundary between the groups on 20 RBC counts /HPF, according to our previous report (7).

### 3.2. Treatment protocol

Patients were treated with 0.8 mg/kg body weight/day oral prednisolone for 1 month. The oral prednisolone was gradually tapered, at a rate of 2.5–5 mg/month, with all patients treated for at least 2 years. Inhibitors of the renin angiotensin system (RAS-inhibitors) were added individually.

### 3.3. Evaluation of clinical and laboratory data

Factors compared in the two groups at the time of renal biopsy included sex, age, percentage of patients with episodes of macrohematuria, interval from onset of IgAN, body mass index, systolic blood pressure (SBP), diastolic blood pressure (DBP), serum total protein, serum albumin, serum creatinine, eGFR, serum total cholesterol, serum IgA, C3, proteinuria, and U-RBC. Serial changes in U-RBC, proteinuria, and eGFR were evaluated for 2 years after renal biopsy.

### 3.4. Histological analysis of renal biopsy specimens

The histological findings were graded according to the Oxford classification (8,9). Four key pathological features were evaluated in each specimen: (*i*) mesangial hypercellularity score (M0, if >50% of glomeruli had fewer than three cells per mesangial area; or M1, if >50% of glomeruli had more than three cells per mesangial area); (*ii*) segmental glomerulosclerosis (S0, absence; or S1, presence); (*iii*) endocapillary hypercellularity (E0, absence; or E1, presence); and (*iv*) ratio of tubular atrophy to interstitial fibrosis in the entire interstitium (T0, 0–25%; T1, 26–50%; T2, >51%). Biopsies containing fewer than eight glomeruli were excluded from analysis.

### 3.5. Ethical issues

1) The research followed the tenets of the Declaration of Helsinki; 2) All patients were included after informed consent; 3) Participation in this study was voluntary and patients were thus free to withdraw from the study at any time without having any effect on their treatment process; 4) The research was approved by the ethical committee of Tokyo Women’s Medical University (#3074).

### 3.6. Statistical analysis

Normally distributed data are presented as means ± standard deviation and analyzed by unpaired *t* tests, and non-normally distributed data are presented as medians ± interquartile range and analyzed by Mann-Whitney U test. The χ^2^ test was used to compare histological grades, sex distribution, episodes of macrohematuria, and administration of RAS-inhibitors. Pairwise correlations among U-RBC, proteinuria, eGFR and histological lesions were assessed using Pearson correlation test. The 20-year renal survival rate was evaluated by the Kaplan-Meier method and the log-rank test. The univariate and multivariate Cox regression analysis were used to identify factors associated with progression to ESRD, with the results expressed as hazard ratios (HR) with 95% CI. Statistical analyses were performed using JMP 10.0.2 software (SAS Institute, Cary, NC, USA), with *P* values < 0.05 considered statistically significant.

## 4. Results

### 4.1. Clinical findings and histological findings according to the Oxford classification

Clinical findings at the time of renal biopsy are shown in Table 1. Sex, age, S-BP, D-BP, the frequency of hematuria and median interval from onset were similar in the two groups. Mean eGFR (69.9±23.4 versus 71.8±21.8 mL/min) and median proteinuria (2.10 [1.34–4.32] versus 2.24 [1.6–3.41] g/day) in the L-RBC and the H-RBC group were also similar. Absolute U-RBC was significantly higher in the H-RBC group (*P*<0.0001). The number of patients who received RAS-inhibitors was also similar between the groups.

**Table 1:** Clinical findings at the time of renal biopsy in the L-RBC and H-RBC groups

	**L-RBC group**	**H-RBC group**	**P value**
Sex (male/female)	10/10	24/31	0.6244
Age (years)	36.7±14.1	30.7±12.5	0.0811
SBP (mm Hg)	126.4±22.8	124.8±15.0	0.7370
DBP (mm Hg)	79.1±17.6	78.6±13.1	0.8896
Macro-hematuria (+/-)	0/20	7/48	0.2199
Interval from onset (years)	4.0 (1.0–12.5)	3.0 (1.0–8.0)	0.5142
Total protein (g/dL)	6.0 (5.6–6.6)	6.2 (5.5–6.7)	0.5547
Serum albumin (g/dL)	3.3 (2.9–4.0)	3.6 (3.1–4.0)	0.3668
Serum creatinine (mg/dL)	0.82 (0.67–0.96)	0.78 (0.69–0.98)	0.9377
eGFR (mL/min)	69.9±23.4	71.8±21.8	0.7540
Uric acid (mg/dL)	6.3±0.3	5.9±0.2	0.2382
Total cholesterol (mg/dL)	234.5 (199.0–286.3)	211.5 (182.3–258.3)	0.0896
Triglyceride (mg/dL)	128.0 (84.0–234.0)	115.5 (81.3–186.3)	0.2452
IgA (mg/dL)	302.0 (231.3–490.5)	293.0 (261.0–400.0)	0.8857
C3 (mg/dL)	74.9 (68.5–84.0)	79.0 (67.4–95.5)	0.5517
Proteinuria (g/day)	2.1 (1.34–4.32)	2.24 (1.6–3.41)	0.8951
U-RBC (counts/HF)	10.0 (5.0–10.0)	80.0 (40.0–100.0)	<0.0001
RAS-inhibitors (+/-)	4/16	10/45	0.6235

Abbreviations: SBP, systolic blood pressure; DBP, diastolic blood pressure; eGFR, estimated glomerular filtration rate; IgA, immunoglobulin A; U-RBC, urinary red blood cells; RAS-Inhibitors, inhibitors of renin angiotensin system

The histological findings are shown in Table 2. Of the patients in the L-RBC and H-RBC groups, 35.7% and 65.9%, respectively, were scored as M1; 57.1% and 72.3%, respectively, as E1; 71.4% and 76.5%, respectively, as S1; 35.7% and 36.1%, respectively, as T1; and 14.2% and 12.7%, respectively, as T2. None of these differences was statistically significant.

**Table 2:** Histological findings according to the Oxford classification in the L-RBC and H-RBC groups

** **	**L-RBC group**	**H-RBC group**	**P value**
Mesangial hypercellularity (M0/M1)	9/5	16/31	0.0872
Endocapillary hypercellularity (E0/E1)	6/8	13/34	0.6107
Segmental glomerulosclerosis (S0/S1)	4/10	11/36	0.9676
Tubular atrophy/Interstitial fibrosis (T0/T1/T2)	7/5/2	24/17/6	0.9900
Out of evaluation	6	8	

### 4.2. Correlations of U-RBC, proteinuria and eGFR with histological findings

Table 3 shows the correlations of U-RBC, proteinuria, and eGFR with histological findings. Proteinuria was correlated slightly and positively with chronic lesions, such as global sclerosis, segmental sclerosis, and fibrous crescent (r=0.28, *P*=0.0175). In addition, eGFR was slightly and negatively correlated with global sclerosis (r=-0.31, *P*=0.0071) and chronic lesions (r=-0.34, *P*=0.0033). U-RBC, however, was not correlated with active glomerular lesions such as cellular and fibro-cellular crescents.

**Table 3:** Correlations between histological findings and U-RBC, proteinuria, and eGFR

	**%**	**U-RBC**		**Poteinuria**		**e-GFR**	
		**r**	**P value**	**r**	**P value**	**r**	**P value**
Global sclerosis	15.4±14.9	-0.18	NS	0.23	NS	-0.31	0.0071
Segmental sclerosis	5.6±6.8	-0.16	NS	0.06	NS	-0.09	NS
Fibrous crescents	3.7±7.8	0.04	NS	0.19	NS	-0.16	NS
Chronic lesions	24.8±19.4	-0.19	NS	0.28	0.0175	-0.34	0.0033
Cellular crescents	6.8±9.8	0.04	NS	-0.13	NS	0.002	NS
Fibro-cellular crescents	5.7±11.1	0.13	NS	-0.09	NS	-0.08	NS
Active lesions	12.7±13.7	0.11	NS	-0.15	NS	-0.07	NS

Abbreviations: U-RBC, urinary red blood cells; eGFR, estimated glomerular filtration rate.

### 4.4. U-RBC, proteinuria and eGFR at 1 and 2 years after steroid therapy and renal survival until ESRD

Table 4 showed proteinuria, U-RBC and eGFR at 1 and 2 years after renal biopsy. Median proteinuria decreased soon after starting steroid treatment, remaining below 1.0 g/g creatinine or g/day at 1 and 2 years in both groups. Proteinuria at the each year was similar between the two groups. Median U-RBC also decreased soon after starting steroid therapy. It was significantly higher in H-RBC group than in L-RBC group (*P*=0.0028) at 1 year after steroid treatment, but it became similar at 2 years. The eGFR was decreased in the two groups, but eGFR at the each year was similar between the both groups.

**Table 4:** Median U-Prot levels, median U-RBC counts, and mean e-GFR over time in the H-RBC and L-RBC groups

	**Baseline**	**1 year**	**P value**	**2 years**	**P value**
**Proteinuria**					
L-RBC group	2.1 (1.34–4.32)	0.66 (0.28-1.63)	0.4770	0.76 (0.22-1.42)	0.9157
H-RBC group	2.24 (1.6–3.41)	0.81 (0.48-1.77)		0.65 (0.05-1.74)	
**U-RBC**					
L-RBC group	10.0 (5.0–10.0)	3.0 (1.0-10.0)	0.0028	4.0 (0.4-10.0)	0.1883
H-RBC group	80.0 (40.0–100.0)	10.0 (4.0-20.0)		6.0 (1.0-20.0)	
**e-GFR**					
L-RBC group	69.9±23.4	61.9±15.9	0.7621	57.3±22.3	0.4202
H-RBC group	71.8±21.8	65.2±26.8		67.4±29.6	

Abbreviations: U-RBC, urinary red blood cells; eGFR, estimated glomerular filtration rate.

**Figure 1:**
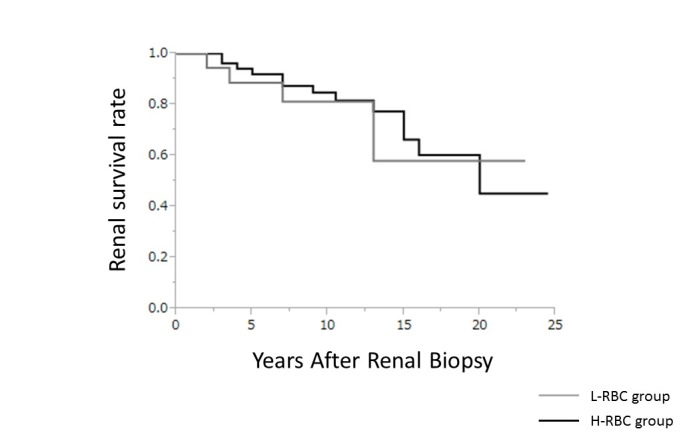
Cumulative renal survival rate in the H-RBC and L-RBC groups.

Figure 1 shows the 20-year renal survival rates until development of ESRD. The cumulative renal survival rate was similar in the L-RBC and H-RBC groups (58.0% versus 45.2%, *P*=0.5577).

### 4.5. Univariate and multivariate analysis of factors associated with ESRD

Table 5 shows the results of a univariate and multivariate Cox regression analysis to identify independent risk factors associated with progression to ESRD. In multivariate analysis, lower eGFR (HR: 2.39, 95% CI: 1.29–4.51, *P*=0.006) was an independent risk factor for disease progression.

**Table 5:** Univariate and multivariate Cox regression analysis of factors independently prognostic for ESRD

	**Univariate analysis**			**Multivariate analysis**		
	**HR**	**95% CI**	**P value**	**HR**	**95% CI**	**P value**
Male (vs. female)	1.28	0.52-3.24	0.587			
Age (10-year increase)	1.20	0.83-1.67	0.325			
Mean BP (10 mm Hg increase)	0.97	0.65-1.43	0.870			
e-GFR (20 mL/min decrease)	2.38	1.45-3.97	0.001	2.39	1.29–4.51	0.006
Proteinuria (1 g/day increase)	1.01	0.85-1.13	0.850			
U-RBC (20/HPF increase)	0.75	0.55-1.00	0.0529			
Mesangial hypercellularity (vs. M0)	2.05	0.71-7.35	0.190			
Endocapillary hypercellularity (vs. E0)	0.48	0.18-1.31	0.148			
Segmental glomerulosclerosis (vs. S0)	1.93	0.62-8.48	0.278			
Tubular atrophy / Interstitial fibrosis (1 grade increase)	2.49	1.19-5.30	0.017	1.78	0.85–3.83	0.1241

Abbreviations: U-RBC, urinary red blood cells; eGFR, estimated glomerular filtration rate.

## 5. Discussion

Hematuria and proteinuria are the most common clinical manifestations of IgAN. Proteinuria is an important risk factor and the best surrogate marker of progression to ESRD (10-12). IgAN patients with nephrotic syndrome have a poorer prognosis than those without nephrotic syndrome (13,14). Although severe hematuria was not found to be a risk factor for progression to ESRD in patients with IgAN (10-12), mild hematuria (U-RBC ≤30/HF) was (15,16). We previously reported that severe hematuria did not have any relationship with increasing proteinuria and progression to ESRD, and naturally decreased in IgAN with mild proteinuria (7). On this study, we found that hematuria did not have the effect on the clinical and histological findings, response against the steroid treatment and outcomes. Moreover, the degree of hematuria did not correlate with active glomerular lesions. Considering our previous report, hematuria did not have any negative impact on the progression, treatment, and prognosis in whole IgAN patients.

Recently, several studies reported the importance of hematuria, however it has been still controversial. Studies from Asia describing the natural course of IgAN with hematuria and minimal proteinuria found that microhematuria at the time of renal biopsy was a risk factor for increased proteinuria, hypertension, and progression to ESRD (17-19). In addition, they emphasized that a higher U-RBC count may indicate a high degree of glomerular inflammation (17,18,20). To date, however, the degree of hematuria has not been found to correlate with the severity of glomerular inflammatory lesions, and our results showed no correlation between U-RBC counts and crescent formation. Gutiérrez et al also reported that the long term prognosis of Caucasian patients of IgAN with normal renal function, microscopic hematuria and mild proteinuria (proteinuria <0.5 g/day) is favorable (21). They suggested that these opposite results from Asia depended on the difference of genetic back grounds, and they also found the agreement point was the percentage of glomerular sclerosis predicted the poor outcome in those IgAN patients with mild proteinuria (19). Indeed, in these reports, one of the factors to reach clinical remission was small number of segmental sclerosis, but endocapillary proliferation as inflammation. Liu et al also reported that the most frequent histological pattern in IgAN patients with isolated hematuria and mild proteinuria (0.5 g/day) was reported to be focal segmental glomerular sclerosis (52.22%), but not crescent formation (24.44%) (22). Interestingly, repeat renal biopsies to evaluate the persistent hematuria from patients in ANCA-associated vasculitis, characterized mainly by hematuria rather than proteinuria, did not have regions of active vasculitis, but had global or focal segmental sclerosis (23,24). These results suggested that an increased number of erythrocytes did not necessarily reflect a high degree of general glomerular inflammation, but may reflect some degree of focal segmental sclerosis. Moreover, mild hematuria was recognized as one of the risk factors for progression from 2450 IgAN patients in multi center from Japan nationwide survey (15,16). Thus, taken together, our and previous results indicate that persistent hematuria may represent not only inflammation of glomerular capillaries but also segmental sclerosis, and that the degree of hematuria does not correlate absolutely with the degree of either glomerular inflammation or segmental sclerosis.

## 6. Conclusions

In conclusion, a higher degree of hematuria at biopsy was not associated with resistance to steroid treatment or poor outcome in IgAN patients presenting with proteinuria >1 g/day, indicating that the states of hematuria and proteinuria differ in patients with IgAN. Hematuria does not reflect absolutely the active inflammation of capillaries and the severity of nephropathy.

## 7. Limitations of the study

Our study had several limitations, including its retrospective observational design and small sample size. Although the clinical and histological backgrounds of the two groups were similar, age tended to be lower and the frequency of patients with mesangial hypercellularity tended to be higher in the H-RBC than in the L-RBC group. After adjusting those clinical findings by propensity score, the survival rate was not significant different between both groups (68.8% in H-RBC group versus 39.0% in L-RBC group, *P*=0.1027). However, the number of patients was smaller by that adjustment (n=12), these biases should be avoided by large, randomized, controlled studies.

## Authors’ contribution

CI was the main investigator, collected the data, and wrote the first draft. TM and KT designed the study, and collected the data. TM was the study supervisor and contributed to the writing process and analysis. TT and KN was the study supervisor, contributed to all aspect of the study, and provided the final manuscript. All authors read and approved the paper.

## Conflicts of interest

Authors declare no competing interests.

## Funding/Support

None.
